# Effect of Chitosan Irrigant and Lubricating Gel on Bond Strength of Resin Sealer to Radicular Dentin: An In Vitro Study

**DOI:** 10.7759/cureus.60143

**Published:** 2024-05-12

**Authors:** Karthika K Kumar, Veena Pai, SN Joshi, Ashok, Roopa Nadig

**Affiliations:** 1 Conservative Dentistry and Endodontics, Dayananda Sagar College of Dental Sciences, Bengaluru, IND; 2 Research and Development, Everest Biotech, Bengaluru, IND

**Keywords:** sealers, bond strength, edta, chitosan, smear layer

## Abstract

Background: The adhesive strength of sealers to dentin is influenced by various factors, and the presence of a smear layer is among the critical variables. Chitosan, known for its dentin compatibility, has previously demonstrated a reduction in dentin change and resin sealer bond strength comparable to ethylenediaminetetraacetic acid (EDTA) when used as an irrigant and final rinse. The study investigates the impact of chitosan, used as both a lubricating gel and final rinse, on the push-out bond strength of resin sealer.

Materials and method: Forty single-rooted premolar teeth, each with a fully formed root and a single root canal, were collected post-extraction. During canal preparation, 1 ml sodium hypochlorite (3%) was used for irrigation at every change of instrument, followed by applying specific chelating gel and final rinse for each experimental group. The groups included: Group 1 (17% EDTA chelating gel, final rinse with saline), Group 2 (17% EDTA chelating gel, final rinse with 17% EDTA solution), Group 3 (chitosan chelating gel, final rinse with saline solution), and Group 4 (chitosan chelating gel, final rinse with 0.2% chitosan solution), 10 specimens in each group. After obturation, specimens were sealed and incubated for a week at 37°C with 100% humidity. The universal testing machine was used for push-out tests, and specimens were examined using a scanning electron microscope (SEM) to identify various types of bond failure.

Results: Among the four groups, Group 2 exhibited the highest mean push-out bond strength (7.33 ± 0.26 MPa), followed by Group 4 (5.33 ± 0.25 MPa), Group 1 (4.61 ± 0.30 MPa), and Group 3 (2.94 ± 0.32 MPa). The variations in bond strength suggest a notable impact of the chelating agents and final rinse solutions on the resin sealer's interaction with dentin.

Conclusion: The study concludes that the use of EDTA as both a lubricating gel and a final rinse significantly enhances push-out bond strength, outperforming chitosan in this study. Groups with saline as the final rinse (Group 1 and Group 3) exhibited the least bond strength, highlighting the importance of the final rinse in root canal therapy.

## Introduction

Necrotic pulp tissue, bacterial biofilms, and their byproducts must be eliminated from the entire root canal complex for effective root canal therapy [1]. The current instrumentation procedures, particularly rotary instrumentation create a smear layer that envelops the root canal walls and closes off the dentinal tubule apertures. The smear layer is made up of two layers: a superficial layer that is 1 to 2 µm thick, and a deeper layer that is 40 µm thick which contains both organic and inorganic materials, including bits of necrotic material, microorganisms, and odontoblastic processes [2].

Sealers are developed to reduce leakage and enhance sealing by penetrating dentinal tubules and accessory canals, allowing radicular dentin and obturating materials to adhere more effectively [3]. Several factors affect the adhesion of sealants to dentin, including dentin intermolecular surface energy, sealant surface tension, and wetting ability that can alter adhesion [4].

Chitosan is a polysaccharide that naturally occurs, and because it is non-toxic, biocompatible, ecological, and bioadhesive in nature, it has attracted interest in dentistry research [5-7] Among the many sealants used for root canal treatment, AH Plus (Dentsply Sirona, NC, USA) sealant has gained popularity due to its ability to improve dentin wettability, and gutta-percha sealability with adequate biological performance [8,9].

It has been demonstrated that using ethylenediaminetetraacetic acid (EDTA) and sodium hypochlorite together makes the surface hydrophobic and improves the adherence of AH Plus (Dentsply) sealers [10]. According to a study, the push-out method was more accurate and dependable than the micro-tensile method for determining bond strength to dentin [11]. Chitosan when used as an irrigant and final rinse has shown less dentin alteration and bond strength to resin sealer like that of EDTA. In all these studies, chitosan is used either as an irrigant or final rinse, but the lubricating agent used for instrumentation was EDTA. This study aimed to assess the impact of chitosan as a lubricating gel and final rinse on the push-out bond strength of resin sealer.

## Materials and methods

**Figure 1:**
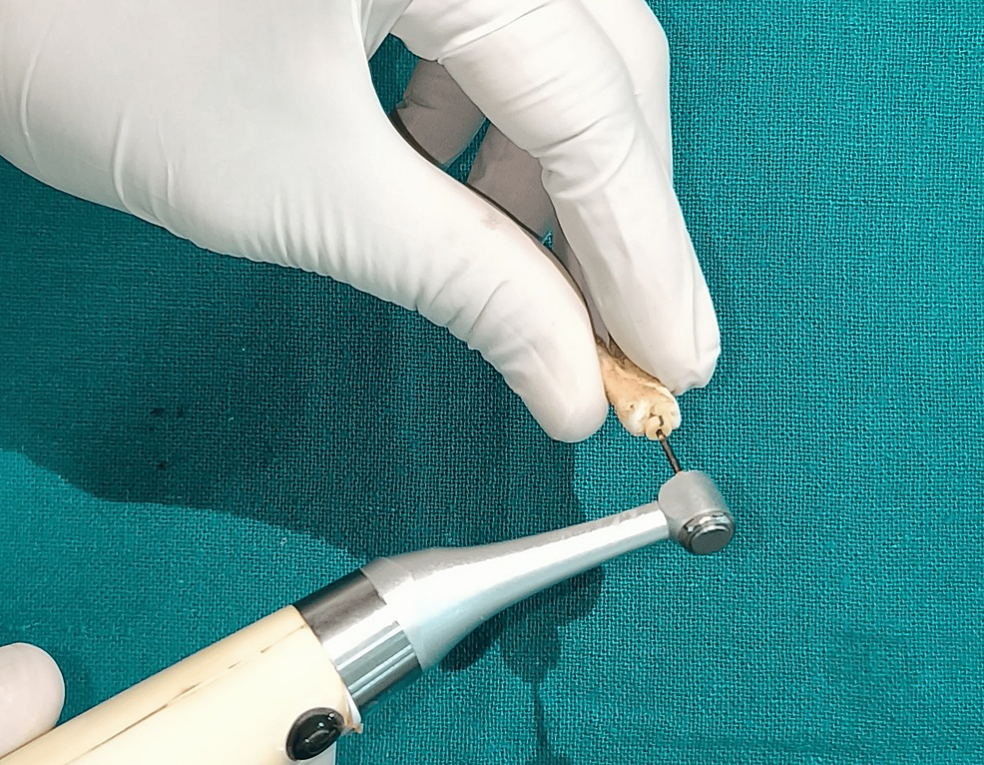
Cleaning and shaping with lubricating gel.

Forty single-rooted premolars with complete root formation and a single canal were selected for this study. Exclusion criteria comprised teeth with caries, anatomical variations, internal resorption, and those previously restored or endodontically treated. The teeth underwent meticulous cleaning and were decoronated at the cementoenamel junction, leaving a 15 mm root length. The working length was determined by subtracting 1 mm from this measurement, and canal preparation was performed using Protaper files up to F3 (Dentsply) (Figure 1). At each instrument change, sodium hypochlorite irrigation was accompanied by a specific chelating gel and final rinse based on the assigned group.

**Figure 2:**
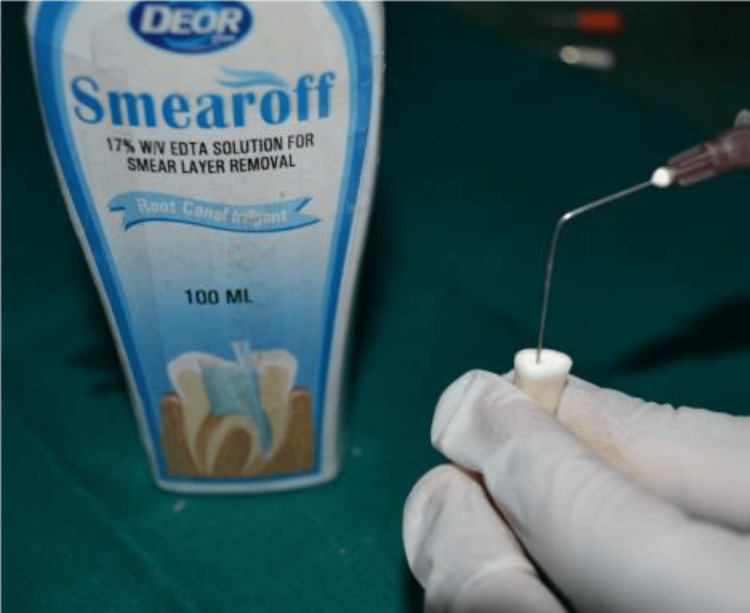
Final rinse with 17% EDTA. EDTA: Ethylenediaminetetraacetic acid.

**Figure 3:**
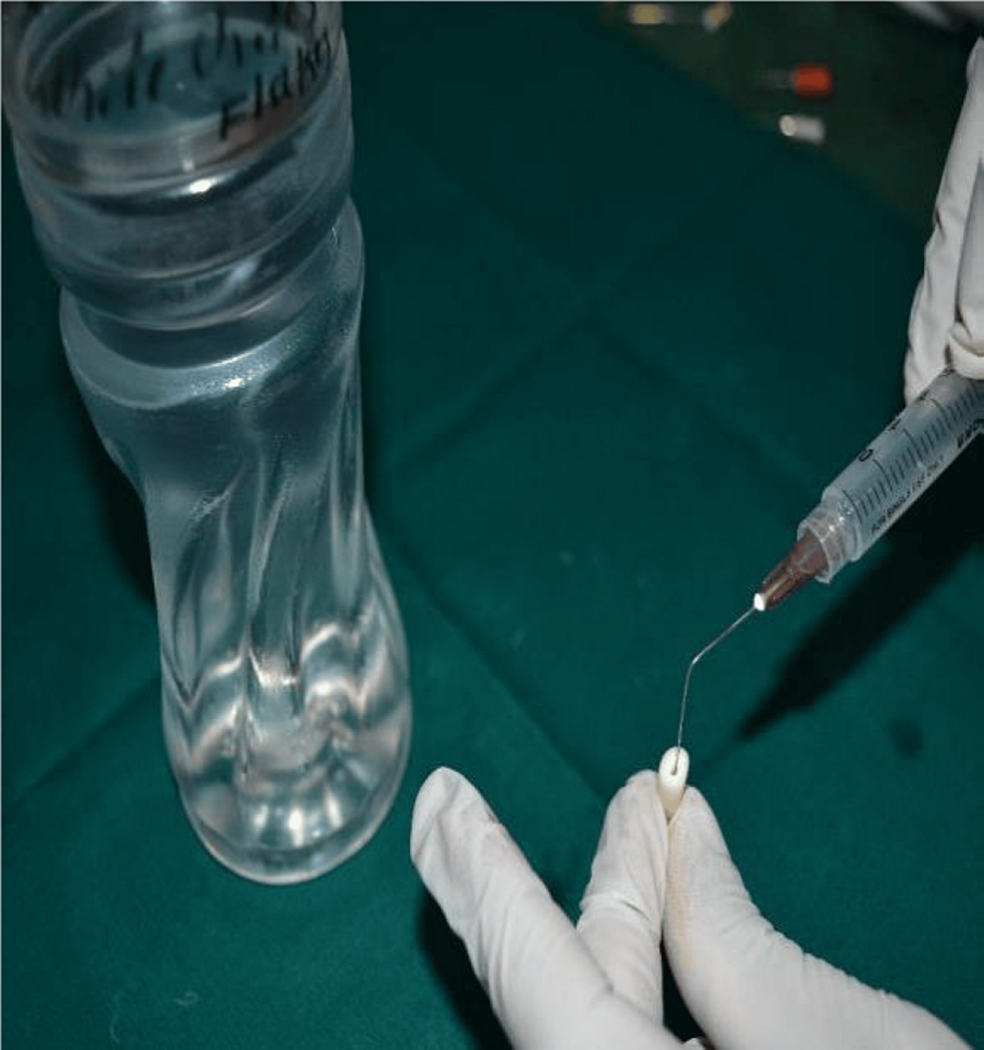
Final rinse with 0.2% chitosan.

The experimental groups were as follows, with 10 specimens in each group: Group 1 (sodium hypochlorite irrigation + 17% EDTA chelating gel + final rinse as saline), Group 2 (sodium hypochlorite irrigation + 17% EDTA chelating gel + final rinse as 17% EDTA solution) (Figure 2), Group 3 (sodium hypochlorite irrigation + chitosan chelating gel + final rinse with saline solution), Group 4 (sodium hypochlorite irrigation + chitosan chelating gel + final rinse with 0.2% chitosan solution) (Figure 3).

EDTA solution (Smearoff) and chitosan solutions were provided by Everest Biotech Research, Bengaluru, India. Following canal preparation, samples were obturated with gutta-percha F3 cones (Dentsply) and AH Plus sealer (Dentsply) where the canal walls are filled with AH Plus (Dentsply) through a pumping or rotating movement in a counterclockwise direction of the instrument. Then alternatively, apply AH Plus (Dentsply) onto the tip of the filing system. Advance the instrument slowly to the apex running at a very low speed. The specimens were sealed and kept at 37°C with 100% humidity for seven days to ensure a complete sealer setting.

**Figure 4:**
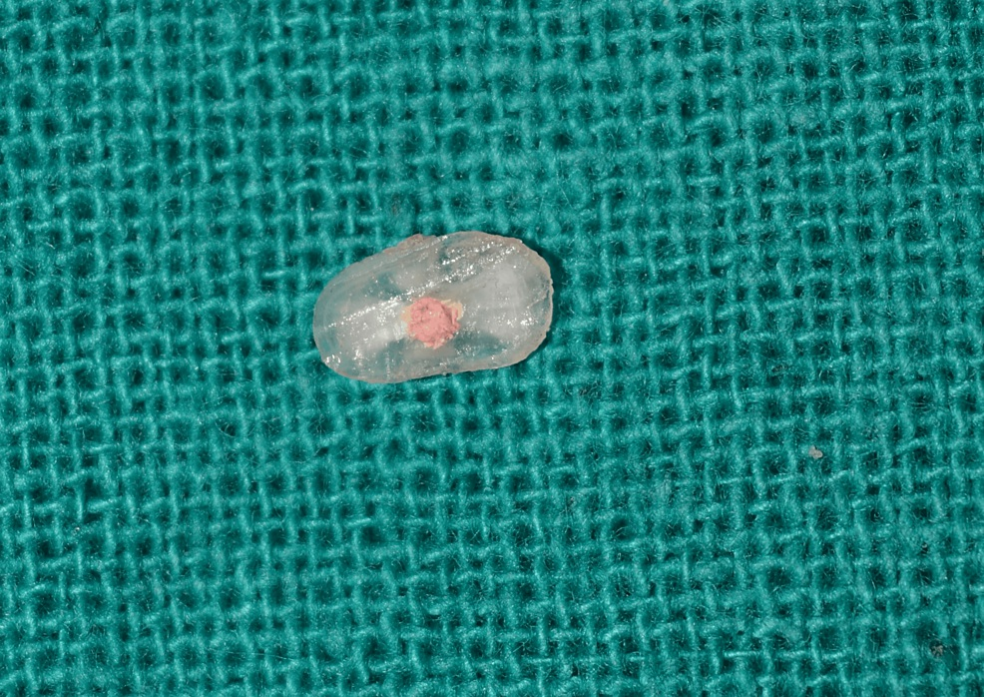
Sample slice used for the test.

**Figure 5:**
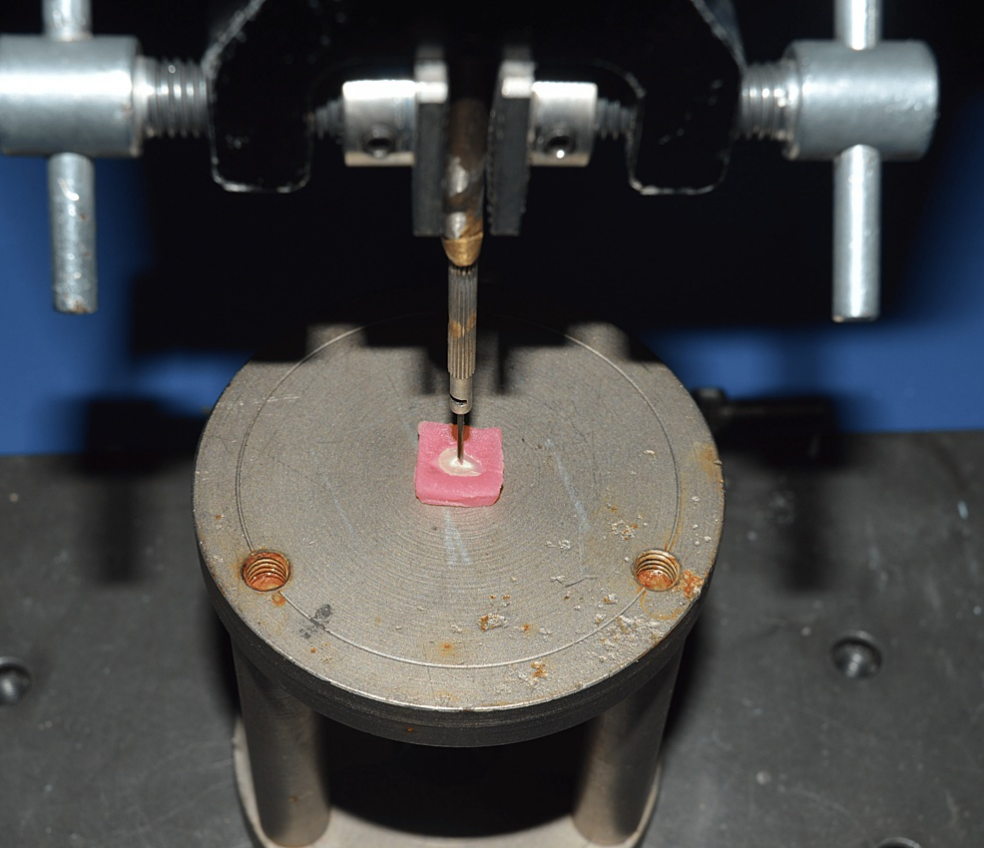
Push-out test for the sample used.

Microtome slices from apical, middle, and coronal specimens were obtained for orientation, and push-out tests were performed using a universal testing machine with a 1 mm/min crosshead speed. Additionally, a subset of samples underwent a scanning electron microscope (SEM) analysis to identify the modes of bond failure post-push-out testing (Figures 4, 5).

This study is carried out by using ANOVA between the groups and the Bonferroni (post-hoc) test for the mean difference between the groups with a 95% confidence interval, the p-value assigned as <0.05.

## Results

The influence of chitosan as an irrigant and lubricating gel on the bond strength of resin sealer to radicular dentin was investigated using one-way ANOVA. When assessing the combined data from all sites (coronal, middle, and apical) across the four groups (1, 2, 3, 4), the mean bond strength was highest in Group 2 (7.33 ± 0.26), followed by Group 4 (5.33 ± 0.25), Group 1 (4.61 ± 0.30), and Group 3 (2.94 ± 0.32). The unit for bond strength is measured in MPa (Tables 1, 2).

**Table 1:** Individual values for coronal, middle, and apical regions.

Region	Group	Minimum (MPa)	Maximum (MPa)	Mean (MPa)	Standard deviation
Coronal	1	4.2	5.0	4.7	0.30
Middle	1	4.1	5.03	4.6	0.31
Apical	1	4.05	4.9	4.5	0.30
Coronal	2	7.0	7.77	7.4	0.26
Middle	2	6.97	7.6	7.3	0.26
Apical	2	6.9	7.5	7.2	0.25
Coronal	3	2.4	3.4	3.0	0.32
Middle	3	2.31	3.2	2.9	0.32
Apical	3	2.3	3.0	2.8	0.31
Coronal	4	4.9	5.7	5.4	0.25
Middle	4	4.87	5.6	5.3	0.25
Apical	4	4.8	5.5	5.2	0.24

**Table 2:** Mean bond strength among all groups.

(Coronal+Middle+Apical)	Minimum	Maximum	Mean (MPa)	Standard deviation
Group 1	4.05	5.031	4.61	0.309
Group 2	6.97	7.77	7.33	0.26
Group 3	2.31	3.42	2.94	0.32
Group 4	4.87	5.7	5.33	0.25

The ANOVA analysis for coronal, middle, and apical regions showed a significant overall difference among the four groups (F=388.16; p=0.000). Post-hoc analysis using the Bonferroni test identified statistically significant differences between all group pairs (p<0.05). The ANOVA findings and the p-value are shown in Table 3 and Table 4.

**Table 3:** One-way ANOVA used for estimating bond strength. * Significant.

(Coronal+Middle+Apical)	F	p-value
Between groups	388.167	0.001*

**Table 4:** Post-hoc analysis using the Bonferroni test for mean bond strength between the groups. * Significant.

Groups	Groups	Mean bond strength (MPa)	p-value	95% Confidence interval	
				Lower bound	Upper bound
1	2	-2.72	0.000*	-3.08	-2.35
	3	1.66	0.000*	1.29	2.02
	4	-0.72	0.000*	-1.08	-0.35
2	3	4.38	0.000*	4.02	4.75
	4	2	0.000*	1.63	2.36
3	4	-2.38	0.000*	-2.74	-2.02

The adhesive type of bond failure was seen in Groups 1 and 3 which used saline as their final rinse. Other test subjects showed a tendency for mixed failure (adhesive and cohesive failure) (Table 5).

**Table 5:** Failure patterns of tested groups under SEM. * Number of teeth denoting failure. EDTA: Ethylenediaminetetraacetic acid; SEM: Scanning electron microscope.

Groups	Adhesive*	Cohesive*	Mixed*
17% EDTA gel + saline	4	4	12
17% EDTA gel + 17% EDTA solution	2	5	13
0.2% chitosan gel + saline	5	2	13
0.2% chitosan gel + 0.2% chitosan solution	2	5	13

## Discussion

In endodontic practice, a variety of root canal sealers are employed, each with its own benefits and drawbacks. Sealers are primarily chosen for their effectiveness in sealing, adhesion, biocompatibility, and antimicrobial resistance.

In this study, AH Plus (Dentsply) sealer was chosen for its renowned ease of handling, sealability, and favorable biological performance in endodontic procedures [8]. The micro-retention of radicular dentin by AH Plus (Dentsply) contributes to enhanced adhesion to obturating materials compared to other sealers [12,13]. The selection of appropriate irrigants is crucial for effective biomechanical preparation, with the smear layer removal being contingent on factors such as irrigant volume, contact time, and concentration. Sodium hypochlorite (NaOCl) served as the standard irrigating solution due to its lack of tissue-dissolving properties and its ability to reduce dentin microhardness, facilitating instrumentation in narrow, calcified root canals [14,15]. The combination of NaOCl with EDTA has been a longstanding and widely adopted approach for effective decalcification [16].

Chitosan, identified as a biocompatible material with chelating properties, demonstrated comparable effectiveness to 17% EDTA in our study [17]. Its versatility in various physical forms, such as film, fiber, beads, powder, or nanoparticles, makes it a promising option for endodontic applications. The success of chitosan as an irrigant, showing efficient smear layer removal without causing structural or chemical changes to radicular dentin, further supports its potential utility in endodontic practice.

Among the examined groups, Group 2, employing EDTA as both the chelating gel and final rinse, exhibited the highest bond strength. In contrast, Group 4, utilizing chitosan for both functions, demonstrated a lower bond strength compared to Group 2 but still outperformed Groups 1 and 3, which employed saline as the final rinse and EDTA and chitosan as chelating agents, respectively [18]. The observed decrease in bond strength in Group 4 may be attributed to the shorter contact duration of the chitosan final rinse. Additionally, the synergistic effects of the final rinse and EDTA gel in removing smear layers, coupled with a decrease in the calcium phosphate ratio, likely contributed to the enhanced push-out bond strength observed in Group 2 [17]. 

This study has some limitations, one is its in-vitro nature. Other teeth were not involved in this study. Future studies should be carried out using different concentrations of irrigants at different time intervals and their effect in vivo.

## Conclusions

Our study concluded that the group that used ethylenediaminetetraacetic acid (EDTA) as a lubricating gel and final rinse had higher bond strength followed by chitosan gel and final rinse. Groups 1 and 3 that used saline as the final rinse had the weakest bonds, highlighting the need to use a final rinse during root canal therapy. The bond failure pattern seen in SEM (scanning electron microscope) showed adhesive failure when saline is used as the final rinse indicating it produces the weakest bonded surface for the sealers. The pattern of bond failures for groups 2 and 4 showed more mixed and cohesive failure, which implies good bonding when both EDTA and chitosan were used as final rinses.
